# Survival in Cervical Cancer and Its Predictors at Ocean Road Cancer Institute From January to December 2012

**DOI:** 10.1200/GO.20.00616

**Published:** 2021-05-19

**Authors:** Salama Iddy Khamis, Alita S. Mrema, Johnson Katanga, Emmanuel L. Lugina

**Affiliations:** ^1^ Ocean Road Cancer Institute, Dar es Salaam, Tanzania; ^2^ Department of Clinical Oncology, Muhimbili University of Health and Allied Sciences (MUHAS), Dar es Salaam, Tanzania

## Abstract

**PURPOSE:**

In Tanzania, cancer of cervix is the most commonly diagnosed cancer and is the leading cause of cancer-related deaths. There are very little data about survival of patients with cervical cancer after treatment in Tanzania. The aims of this study were to determine 5-year overall survival (OS) rate and its predictors among patients with cervical cancer treated at Ocean Road Cancer Institute (ORCI) from January to December 2012.

**MATERIALS AND METHODS:**

This was retrospective study done at ORCI by reviewing medical charts of 202 patients with cervical cancer treated at ORCI from January to December 2012. A structured questionnaire was used to extract information about characteristics of the respondents. Survival curves were estimated by using Kaplan-Meir analysis and were compared by using log-rank test.

**RESULTS:**

The 5-year OS rate was 26%. The mean and median survival times were 33.9 and 19 months, respectively. Factors that were positively associated with 5-year OS rate include the hemoglobin level more than 9 g/dL at presentation, early International Federation of Gynecology and Obstetrics stage at presentation, high dose of radiotherapy, and use of concurrent chemoradiotherapy. Histology type and HIV status were not associated with survival.

**CONCLUSION:**

The 5-year overall survival rate was 26%. Predictors of OS were hemoglobin level, stage at presentation, radiotherapy dose, and the use of concurrent chemoradiotherapy.

## INTRODUCTION

Cervical cancer is a major public health problem. With an estimated 570,000 new cases and 314,000 deaths in 2018 worldwide, this disease ranks as the fourth most frequently diagnosed cancer and the fourth cause of cancer deaths among women. The developing countries constitute 86% of the newly diagnosed cases and 88% of the deaths. Cervical cancer is the most commonly diagnosed cancer in 28 countries including Tanzania and is the leading cause of cancer deaths in 42 countries, the majority of which are in sub-Saharan Africa again including Tanzania.^1^

**CONTEXT**

**Key Objective**
What are the survival outcomes of patients with cervical cancer from resource-limited countries?
**Knowledge Generated**
Survival outcomes have been shown to be poor in resource-limited countries because of poor radiotherapy facilities leading to low doses of radiation to the tumor. Most countries in these settings do not have brachytherapy facilities, and a majority of patients cannot afford chemotherapy. Other factors that contribute to poor survival are advanced stage of disease at presentation and anemia at diagnosis. This study showed an overall 5-year survival rate of 26%. Similar findings have been shown in Uganda.
**Relavance**
There is a need of improving access to radiotherapy facilities especially brachytherapy in treating cervical cancer, improving mass awareness about cervical cancer, strengthening screening services, and implementation of human papillomavirus vaccination in these settings to improve survival.

Over the last few decades, cervical cancer incidence and mortality have been declining in developed parts of the world. These declines have been attributed to screening, increasing average level of social economic level, and diminishing risk of high-risk persistent human papillomavirus infection resulting from improvement in genital hygiene, reduced parity, and diminishing risk of sexually transmitted infections.^2^ In the absence of effective screening as in sub-Saharan Africa, there has a rapid increase in premature cervical cancer mortality in recent generation.^3^

The management of cervical cancer is a major challenge in sub-Saharan Africa, and this has resulted in very high mortality rate.^4^ The 5-year survival rate in sub-Saharan Africa ranges from the highest in Mauritius at 82.1% and lowest in Kampala, Uganda, at 24.0% depending on human development index.^5^

There is little information on survival of patients with cervical cancer in Tanzania. The aims of this study were to determine the 5-year overall survival (OS) rate and its predictors at Ocean Road Cancer Institute in the period January-December 2012.

## MATERIALS AND METHODS

### Ethical Statement

Ethical clearance was sought from Ethical Clearance Board (IRB) of the Ocean Road Cancer Institute.

### Study Design and Population

This was a retrospective descriptive study conducted at Ocean Road Cancer Institute (ORCI). Approximately 4,190 new patients with cancer are seen at ORCI annually, of which approximately 39% have cervical cancer. Medical charts of respondents with cervical cancer who presented to ORCI from January to December 2012 were reviewed.

### Inclusion and Exclusion Criteria

Patients who were above age 18 years and with histologically confirmed cervical cancer were included in the study. Patients with incomplete records were excluded from the study.

### Treatment Protocol

**FIG 1:**
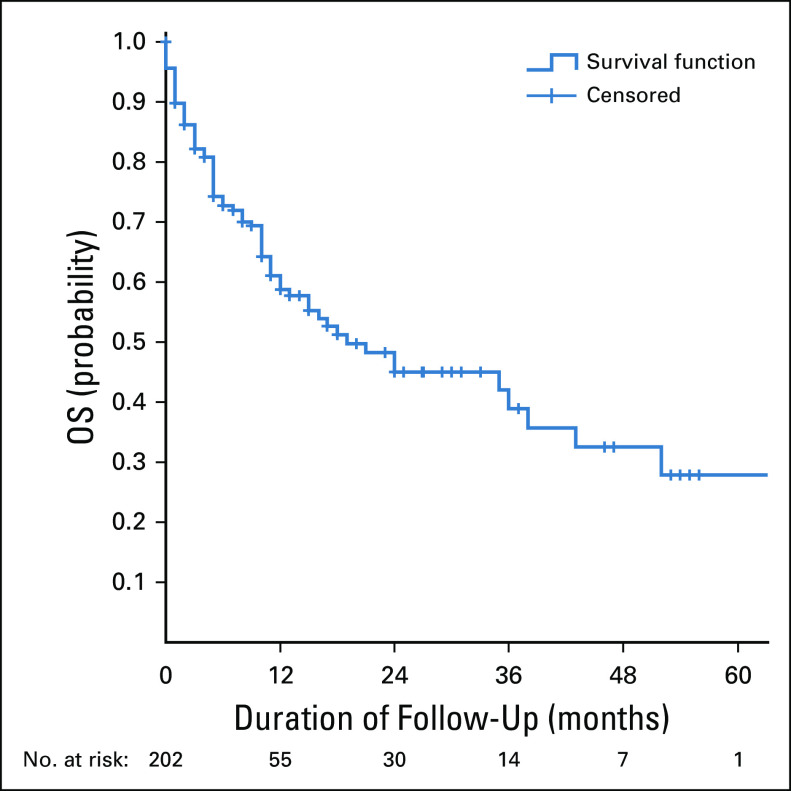
The OS of patients with cervical cancer. OS, overall survival.

**FIG 2:**
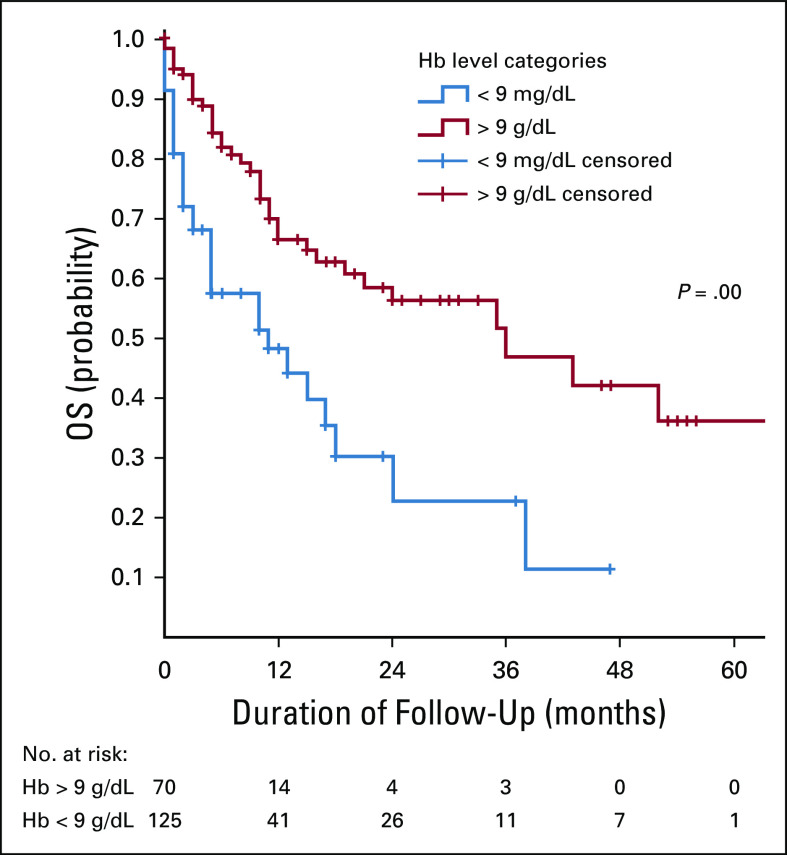
The association between OS rate and Hb level. Hb, hemoglobin; OS, overall survival.

**FIG 3:**
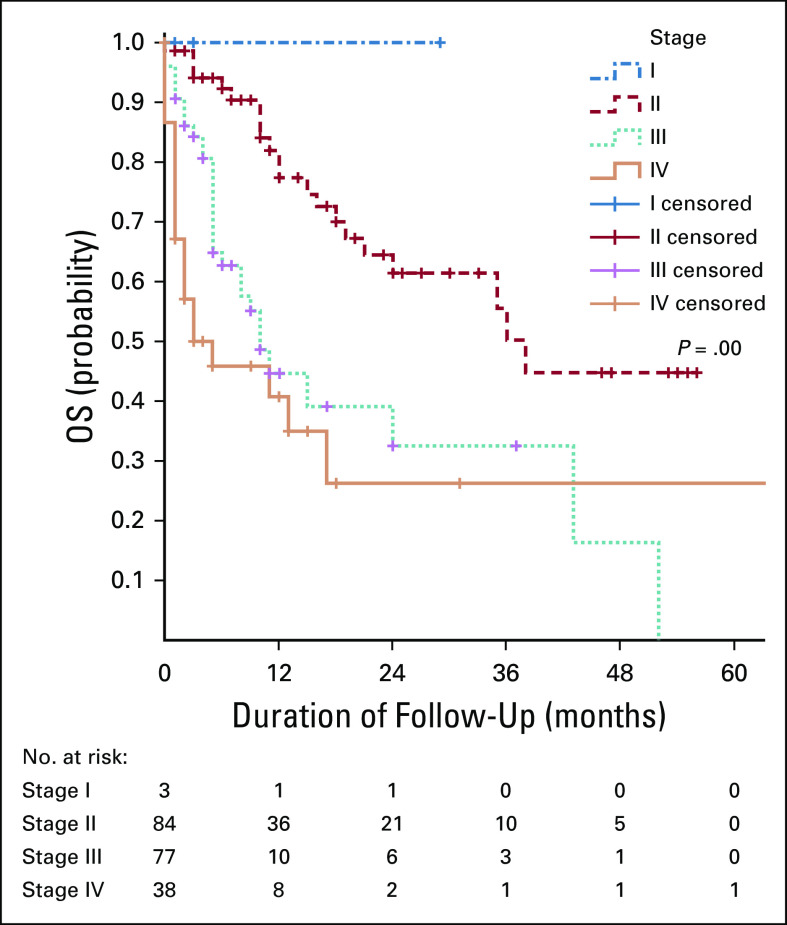
The association between OS rate and FIGO stage. FIGO, International Federation of Gynecology and Obstetrics; OS, overall survival.

All patients had chest x-ray and abdominal pelvic ultrasound before treatment. Assessment of pelvic and para-aortic lymph node metastasis was not performed. Radiation therapy represented the primary therapy for all the patients. Radiation treatment consisted of external beam radiotherapy, two-dimensional technique. Extended field radiotherapy for possible para-aortic lymphadenopathy was not used. Patients were treated with Cobalt-60 photons’ beam energy. The radiation technique applied was the classical technique of conventional consisting of two phases. The first phase referred to the field setup for 50 Gy (25 fractions of 2 Gy/fraction) and was represented by two opposing anteroposterior fields. In the second phase, 14 Gy more than seven fractions was applied to the smaller anteroposterior fields. The 4-field technique was not used because of patient overload. Patients received cisplatin (40 mg/m^2^) once a week for 6-7 weeks, given concurrently with external beam radiotherapy on an outpatient basis. Brachytherapy was not given because it was not available. Patients were followed up once in 3 months for the first 2 years, then once in 6 months in the next 3 years, and then annually for next 5 years. During follow-up physical examination, pelvic examination was also performed and abdominal pelvic ultrasound and chest x-ray were performed to assess locoregional recurrence or distant metastasis. There was no gynecologic oncologist in the country during the study period (Figs 1-3).

### Data Collection and Variables

Data were extracted from medical charts by using a structured questionnaire. The covariates were age, marital status, stage, histology, hemoglobin level, HIV status, and dose of radiotherapy. The OS was calculated from date of finishing radiotherapy to the date of death or loss to follow-up.

### Statistical Methods

SPSS version 21 was used for statistical analysis. Continuous variables were summarized and presented as frequency and mean, whereas categorical variables were summarized as proportions. OS was calculated by Kaplan-Meir analysis. Log-rank test was used to identify predictors of survival. The *P* value of < .05 was considered statistically significant.

## RESULTS

In this study, 202 respondents were included (Table 1). The mean age among the respondents who were HIV-positive and HIV-negative was 44.6 and 54.6 years, respectively. The mean parity of the respondents was 6. Of 84 respondents, who had stage II diseases, 33% had stage IIA and 67% had stage IIB. Of 38 respondents who had stage IV diseases, 27 had stage IVA and 11 had stage IVB.

**TABLE 1:**
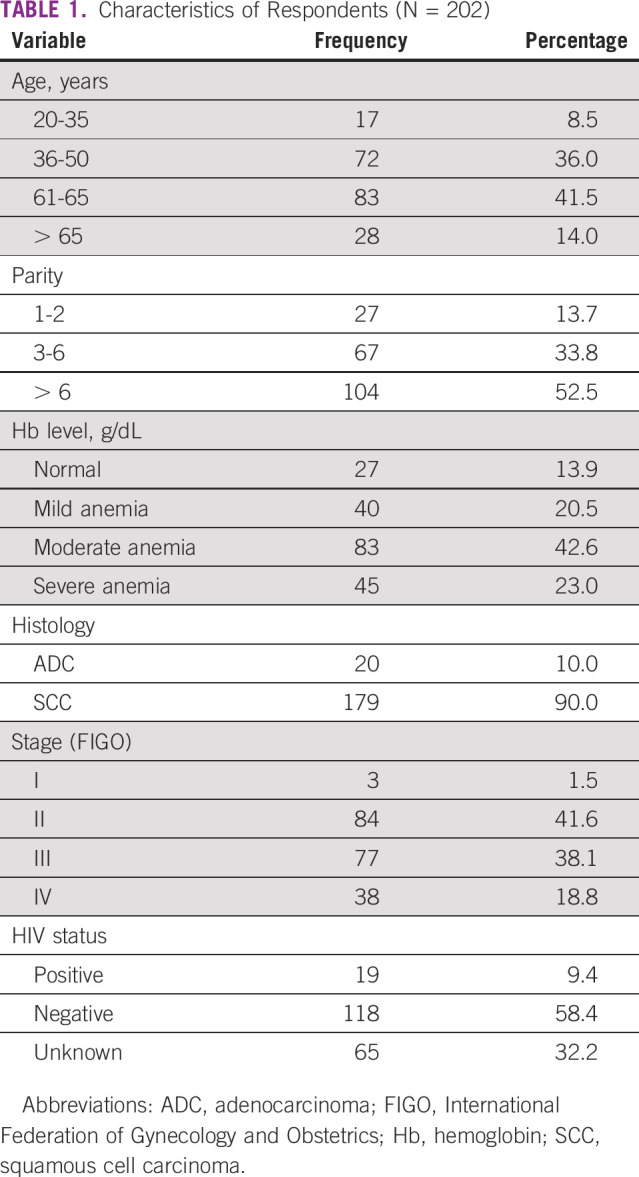
Characteristics of Respondents (N = 202)

**FIG 4:**
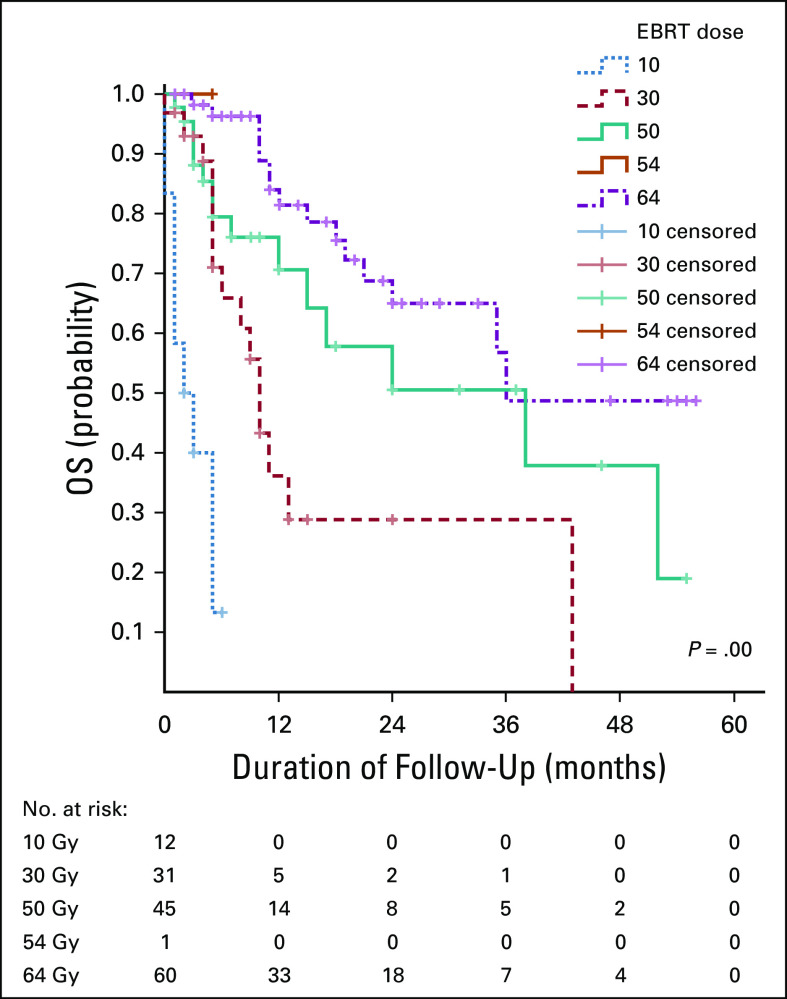
The association between OS rate and radiotherapy dose. EBRT, external beam radiotherapy; OS, overall survival.

**FIG 5:**
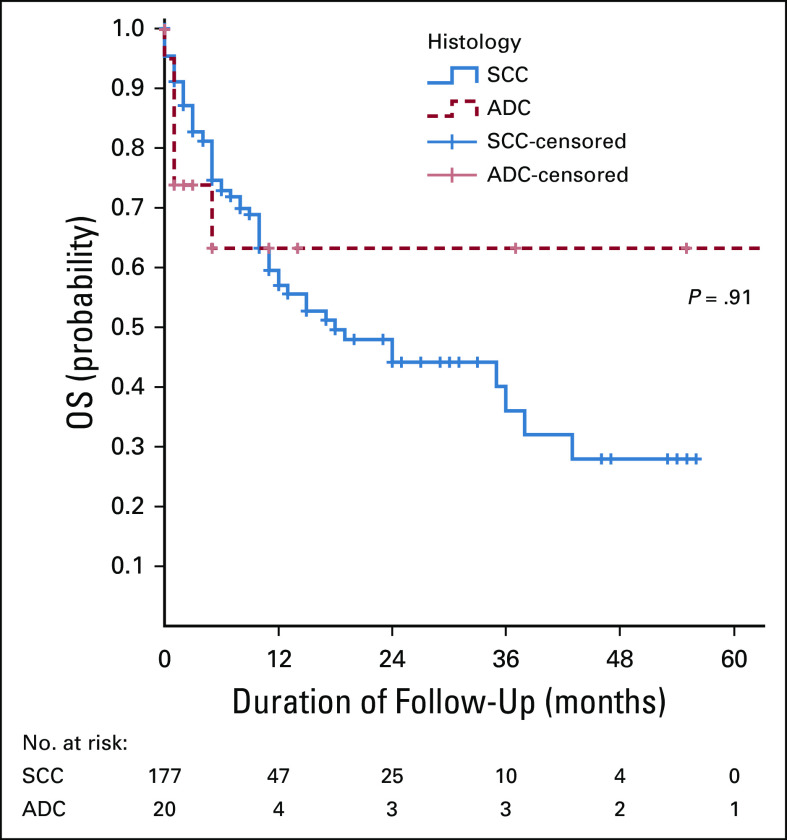
The association between OS rate and type of histology. ADC, adenocarcinoma; OS, overall survival; SCC, squamous cell carcinoma.

**FIG 6:**
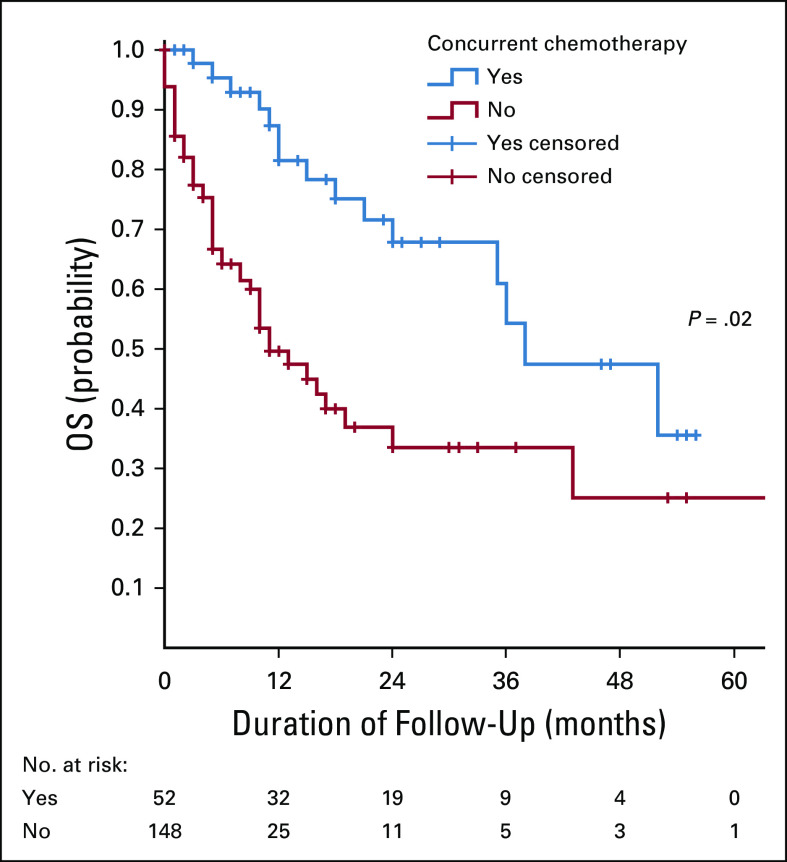
The association between OS rate and the use of concurrent chemoradiotherapy. OS, overall survival.

Of 202 respondents, only 149 were treated by radiotherapy (Table 2). Only 52 respondents of 149 who were treated by radiotherapy received concurrent chemoradiotherapy. The mean total biologically equivalent dose in 2 Gy fractions among those treated with curative and palliative radiotherapy was 54 Gy and 23 Gy, respectively. The mean and median duration of treatment among those treated with curative intent were 39 and 42 days. None of the respondents were treated by surgery or brachytherapy. Fifty-three patients were not given any treatment because of either poor performance status of absconding before treatment. Toxicity data were not available because of poor documentation (Figs 4-6).

**TABLE 2:**
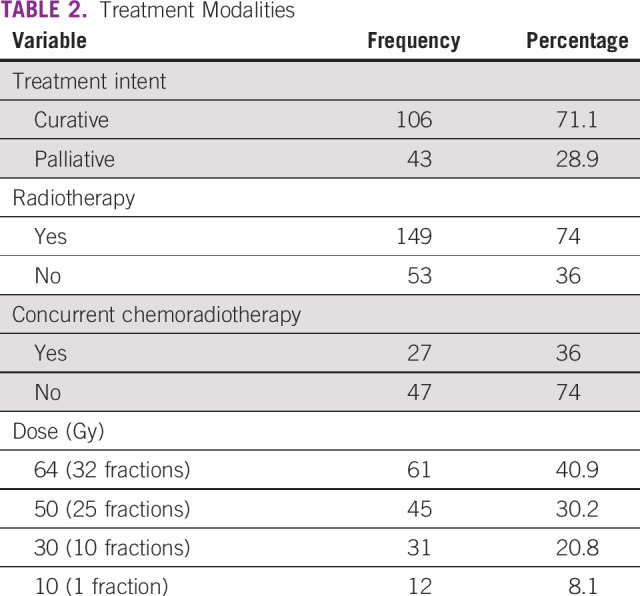
Treatment Modalities

## DISCUSSION

In this series, the mean hemoglobin level was 9.7 g/dL for the entire cohort. Respondents with a hemoglobin level more than 9 g/dL had a higher OS after radiotherapy compared with those with a hemoglobin level < 9 g/dL. Recent data from clinical studies indicate that the relationships between anemia and tumor hypoxia in patients with cancer who are treated by radiotherapy are much more complex than those initially perceived.^6^ Anemia has been linked to tumor hypoxia and therefore tumor radioresistance. Oxygen delivery to the tumor peaks at a hemoglobin concentration of about 11 g/dL and decreases at lower hemoglobin concentrations because of a combination of the reduced oxygen-carrying capacity of the blood and the systemic effects of anemia limiting tumor blood flow.^6^ Anemia is also correlated with advancing tumor stage.^7^ Some authors reported that anemia does not exert an independent prognostic impact among patients with cancer treated by radiotherapy but represents only an epiphenomenon linked to known adverse prognostic factors such as tumor advancement.^6^ In an analysis of patients with advanced disease in Gynecologic Oncology Group trials, anemia did not influence survival.^8^ In contrast, in a study by Kapp et al,^9^ hemoglobin level was shown to be associated with survival. Anemia may be an independent prognosis predictor through increase in fraction of hypoxic tumor cells during radiotherapy, or it may be a surrogate marker of advanced disease via tumor size and nodal metastasis.

In this study, respondents with advanced stages had statistically significant lower OS rate compared with those with early stages. Other studies have reported similar findings.^4,5,10,11^ In Tanzania, human papillomavirus vaccination was launched in 2018. Cervical cancer screening is mainly performed by using visual inspection methods, and very few centers are able to do Pap smear. The government and nongovernmental organizations have been working together to raise awareness about cervical cancer and screening for cervical cancer.

Findings in the index study show that higher radiotherapy dose was associated with a higher OS. The mean total biologically equivalent dose in 2 Gy fractions of radiotherapy to Point A among those treated with curative intent was only 54 Gy. This could be attributed to lack of intracavitary brachytherapy during the study period. Brachytherapy is an integral component of definitive treatment for patients with locally advanced cervical cancer. The steep dose gradient allows for the delivery of highly conformal doses of radiation to the central pelvis, minimizing toxicities and maximizing tumor control. The recommended total biologically equivalent dose in 2 Gy fractions from teletherapy and brachytherapy to point A for locally advanced cervical cancer is above 80 Gy.^12^ In an analysis of 565 patients with various stages of cervical carcinoma treated in the Patterns of Care Study, Coia et al reported better survival (67%) and pelvic tumor control (78%) for patients receiving intracavitary brachytherapy than for patients who had no intracavitary brachytherapy applications, for whom the 4-year survival rate was 36%.^13^ The lack of brachytherapy may be one of the reasons associated with low 5-year OS rate in this series. ORCI has long-term plan to introduce brachytherapy.

Finding from this study showed that there was no association between histology type and OS rate after radiotherapy. Similar findings were observed by Katanyoo et al^14^ although the median time to complete response rate was a little bit longer for adenocarcinoma compared with squamous cell carcinoma. In another study performed by Rose et al,^15^ it was shown that adenocarcinoma and adenosquamous carcinomas of the cervix are associated with worse OS when treated with radiation alone but with similar progression-free survival and OS compared with squamous cell carcinomas of the cervix when treated with concurrent chemoradiotherapy.

The finding from the index study showed that HIV status does not influence survival among patient with cervical cancer after treatment. This finding is not in line with findings from the studies by Ferreira et al in Brazil^16^ and Dryden-Peterson et al in Botswana,^17^ which showed that in the context of good access to and use of antiretroviral treatment, HIV infection significantly decreases cervical cancer survival. Perhaps the lack of association in the index could be attributed to the smaller sample size.

In this series, it was shown that respondents who were treated by concurrent chemoradiotherapy had a higher OS compared with those who were treated by radiotherapy alone. Concurrent chemoradiotherapy has been shown to improve survival compared with radiotherapy alone in RTOG 90-01.^18^

The 5-year OS rate in this study was only 26%. This finding could be due to high prevalence of anemia at diagnosis, advanced stage at diagnosis, lack of brachytherapy, and few patients treated with concurrent chemoradiation. In a study by Anorlu et al, the 5-year relative survival rates in Kampala, Uganda, and Harare, Zimbabwe, in 1990 were 18% and 30%, respectively, whereas during the same period, the rate was 72% in the United States. The causes of high mortality and low survival rates in Africa are poor access to medical facilities (worst in the rural areas); poor nutrition and co-morbid conditions, eg, anemia; HIV infection; late presentation with the disease; large tumor at presentation; poor quality of care provided by many health services; high rate of loss to follow-up; and women not completing treatment because of barriers imposed by poverty. Facilities for treatment are also limited, and where they are available are not affordable to most women in the region.^4^

In conclusion, the 5-year OS rate was 26%. Predictors of OS were anemia, stage at presentation, radiotherapy dose, and use of concurrent chemoradiotherapy, whereas the histology type and HIV status were not associated with survival.
